# Stress-Induced Premature Senescence Promotes Proliferation by Activating the *SENEX* and p16^INK4a^/Retinoblastoma (Rb) Pathway in Diffuse Large B-Cell Lymphoma

**DOI:** 10.4274/tjh.galenos.2019.2019.0117

**Published:** 2019-11-18

**Authors:** Jiyu Wang, Zhitao Wang, Huiping Wang, Zhixiang Wanyan, Ying Pan, Fengfeng Zhu, Qianshan Tao, Zhimin Zhai

**Affiliations:** 1 The Second Affiliated Hospital of Anhui Medical University, Department of Hematology, Hefei, Anhui, P.R. China

**Keywords:** Stress-induced premature senescence, Proliferation, SENEX, p16, Rb/pRb, Diffuse large B-cell lymphoma

## Abstract

**Objective:**

Cellular senescence has been thought to be an important barrier to tumor formation. Recent studies have shown that stress-induced premature senescence (SIPS) can promote partial tumor invasion, but how SIPS affects diffuse large B-cell lymphoma (DLBCL) remains inconclusive. This study aimed to address that issue.

**Materials and Methods:**

The immunophenotype of the LY8 cell line was measured with flow cytometry. SIPS induced by tert-butyl hydroperoxide (tBHP) was detected by senescence β-galactosidase staining. Cell proliferation was analyzed with CCK8 and expression levels of ARHGAP18 (*SENEX* gene-encoding protein), p16/p21, and Rb/pRb were measured with western blot. LY8 cells were transfected with *SENEX*-SiRNA/NC and verified by western blot.

**Results:**

Our results suggested that the immunophenotype of the LY8 cell line is CD19-, CD20-, and CD10-positive and the immunoglobulin light chain is the kappa type. The cellular senescence model of DLBCL could be successfully induced by 30 μM tBHP. ARHGAP18, p21, p16, and Rb protein levels were significantly increased but the level of pRb expression was decreased in the SIPS group compared with other groups. Meanwhile, the proliferation rate was increased in the SIPS group more than other tBHP groups. Furthermore, the expressions of p21 and p16 were significantly decreased in the *SENEX*-SiRNA group compared with the negative control group.

**Conclusion:**

SIPS formation activates ARHGAP18 and the p16/Rb pathway and promotes DLBCL cell proliferation. Furthermore, *SENEX* activates the p16 pathway in DLBCL. SIPS promotes proliferation by activating *SENEX* and the p16/Rb pathway in DLBCL. *SENEX*-related SIPS may serve as an important target for relapsed/refractory DLBCL therapy.

## Abstract (tr)

**Amaç:**

Hücresel yaşlanmanın tümör oluşumuna karşı önemli bir engel olduğu düşünülmektedir. Son çalışmalarda stresin tetiklediği erken yaşlanmanın (senesens) (SIPS) kısmi tümör invazyonunu kolaylaştırabileceği gösterilmiş olmakla birlikte SIPS’nin diffüz büyük B hücreli lenfomayı (DBBHL) nasıl etkilediği bilinmemektedir. Çalışmada bu konunun ele alınması amaçlanmıştır.

**Gereç ve Yöntemler:**

LY8 hücre dizisinin immünfenotipi akış sitometri ile belirlendi. Tert-butil hidroksiperoksitin (tBHP) oluşturduğu SIPS β-galaktozidaz boyası ile tesbit edildi. Hücre proliferasyonu CCK8 ile analiz edildi ve ARGHAP18 (*SENEX* genini kodlayan protein), p16/p21 ve pRb ekspresyon seviyeleri western-blot ile ölçüldü. LY8 hücreleri *SENEX*-SiRNA/NC ile transfekte edilerek western-blot ile gösterildi.

**Bulgular:**

Sonuçlarımıza göre LY8 hücre dizisinin immünfenotipi CD19, CD20 ve CD10 pozitiftir ve immünoglobulin hafif zinciri kappadır. DBBHL’de hücresel yaşlanma modeli 30 μM tBHP ile başarılı bir şekilde oluşturulabilir. ARHGAP18, p21,p16 ve Rb protein seviyeleri anlamlı olarak arttı fakat pRb ekspresyon seviyeleri diğer gruplarla karşılaştırıldığında SIPS grubunda azaldı. Bu arada, SIPS grubunda, diğer tBHP grubu ile karşılaştırıldığında proliferasyon hızı daha çok arttı. Ek olarak, p21 ve p16 ekspresyonları *SENEX*-SiRNA grubunda, negatif kontrol grubu ile karşılaştırıldığında anlamlı ölçüde azaldı.

**Sonuç:**

SIPS oluşumu ARHGAP18 ve p16/Rb yolağını aktive eder ve DBBHL hücrelerinin proliferasyonunu artırır. Ek olarak *SENEX* DBBHL’da p16 yolağını aktive eder. SIPS DBBHL’de *SENEX* ve p16/Rb yolağını aktive ederek proliferasyonu destekler. *SENEX* ilişkili SIPS relaps/refrakter DBBHL tedavisinde önemli bir hedef olabilir.

## Introduction

Diffuse large B-cell lymphoma (DLBCL), representing about 30%-40% of non-Hodgkin’s lymphoma (NHL), is the most common subtype [1]. The introduction of rituximab (R) in combination with standard cyclophosphamide, doxorubicin, vincristine, and prednisolone (CHOP) chemotherapy, known as “R-CHOP”, has significantly improved survival outcomes [2]. However, approximately 30% of cases of advanced-stage DLBCL remain intractable and the disease could relapse in the end [3]. In recent years, cellular immunotherapy has achieved important breakthroughs, especially CD19-chimeric antigen receptor T-cells (CAR-T-CD19) for the treatment of relapsed/refractory acute B lymphoblastic leukemia with up to 70%-90% complete remission rates, but in B-cell lymphomas such as DLBCL, CAR-T treatment did not achieve similar satisfactory results, with only about 50% of the response rate [4]. Studies have suggested that this difference may be related to the specific immune escape protection mechanism of DLBCL [5]. Tumor cell immune escape is associated with the paracrine effects of cellular senescence [6,7,8]. Cellular senescence refers to a relatively stable and continuous state leading to cell detachment from the cell cycle and loss of proliferation during various non-lethal pressures from inside and outside. It is divided into replicative senescence and stress-induced premature senescence (SIPS) according to the different mechanisms [9]. SIPS is telomere-independent and occurs after stimulation by autologous oncogenes, external oxidative and genotoxic substances, or infections. When stress is relieved or the environment changes, SIPS cells may resuscitate, reentering the cell cycle and proliferating [8,10]. Cellular senescence has been thought to be an important barrier to tumor formation. Recent studies have shown that SIPS can promote partial tumor invasion [11].

*SENEX* is a new gene associated with SIPS that was identified as a successful clone in 2004 and was named ARHGAP18 in the RefSeq system [12]. Studies have revealed that *SENEX* can regulate p16^INK4a^ and Rb protein activation in endothelial cells (ECs) under conditions of H_2_O_2_-mediated stress [13]. Once a senescence signal is received from the p53 and p16 pathways, the Rb protein becomes the central link in the control of the aging process. In this study, endogenous *SENEX* remains unchanged during endothelial aging in ECs, but when exposed to oxidative stress, *SENEX* levels are altered, and activated *SENEX* mediates EC SIPS formation and produces resistance through the p16 pathway. Inflammation and *SENEX* overexpression do not alter the expression of p53 or p21. This result suggests that the *SENEX* gene mediates the SIPS mechanism in ECs primarily through the p16 pathway rather than the p53/p21 pathway. However, how does the *SENEX* gene trigger the SIPS phenomenon found in vascular EC functions in tumor cells? Our previous study illustrated that *SENEX* gene expression was upregulated in regulatory T cells (Tregs) of elderly bladder cancer patients, while silencing of the *SENEX* gene by SiRNA increased Treg apoptosis and pro-apoptotic gene expression in response to tBHP-mediated stress [14]. However, the way in which SIPS affects DLBCL remains inconclusive. The present study aims to address this question.

## Materials and Methods

### Cell Culture

Human DLBCL cell line OCI-LY8 was cultured in RPMI (Roswell Park Memorial Institute)-1640 medium supplemented with 10% fetal bovine serum (FBS). Cell cultures were maintained and incubated at 37 °C in humidified air with 5% CO_2_.

### Phenotype Analysis

For analysis of the immunophenotype of the DLBCL LY8 cell line, cells were harvested for flow cytometry (FC-500, Beckman Coulter, Miami, FL, USA). Antibodies were purchased from Beckman Coulter as follows: FITC fluorescently labeled CD19, PE fluorescently labeled CD10, PE fluorescently labeled CD20, ECD fluorescently labeled CD19, FITC fluorescently labeled kappa, and PE fluorescently labeled lambda.

### Induction of Senescence

A tert-butyl hydroperoxide (tBHP) stock solution (5 mol/L) was purchased from Energy Chemical (Shanghai, China). The tBHP stock solution was diluted in RPMI-1640 supplemented with 10% FBS to final concentrations of 10, 30, and 50 µM, and then LY8 cells (10^6^/mL) were treated with 10, 30, or 50 µM tBHP respectively for 24 h in vitro.

### Senescence Staining

According to the Senescence β-Galactosidase Staining Kit (Beyotime, Shanghai, China), LY8 cells treated with 10, 30, and 50 µM tBHP were fixed with galactosidase fixative and incubated in dyeing working fluid. Finally, stained cells were observed under a microscope (CNOPTEC, Chongqing, China). Cells that stained green-blue were evaluated as positive senescent cells.

### SiRNA Synthesis and Transfection

The individual small interfering RNA target *SENEX* gene (*SENEX*-siRNA) and scrambled negative control siRNA (NC) (the sequences are listed in Table 1) were synthesized by Sangon (Shanghai, China). The final siRNA concentration was 33 nM [14]. LY8 cells (4×10^5^/well) were plated in 24-well plates overnight and were then transfected with *SENEX*-SiRNA or NC for 24 h using Lipofectamine 2000 (Invitrogen, Carlsbad, CA, USA) according to the manufacturer’s protocols.

### RNA Extraction and Quantitative Real-Time Polymerase Chain Reaction (qRT-PCR) Analysis

Total RNA was extracted from LY8 cells using TRIzol (Invitrogen, Carlsbad, CA, USA). RNA was reverse-transcribed using a Transcript RT Kit (Sangon, Shanghai, China). qRT-PCR was performed on the ABI 7500 Real-Time PCR System (Life Technologies, Grand Island, NY, USA) using SYBR Green PCR Master Mix (TaKaRa, Dalian, China). All primers were synthesized by Sangon (Shanghai, China). The relative *SENEX* expression level was calculated using the 2^-^^ΔΔCt^ method. Sequences used for qRT-PCR primers and SiRNA transfection are shown in Table 1.

### Western Blot

Total proteins from cells were extracted by western blot with IP cell lysis liquid (Beyotime, Shanghai, China) according to standard procedures (Table 2). Proteins were developed using the SuperSignal West Femto Trial Kit (Thermo Fisher Scientific, Shanghai, China) as previously described [15].

### Proliferation Analysis

LY8 cells were plated at a density of 5000 cells/well in 96-well plates and subsequently transfected with *SENEX*-SiRNA or NC at a final concentration of 33 nM. At 24 h or 48 h after transfection, cell proliferation was measured with the CCK-8 Kit (BestBio, Shanghai, China) [16]. Each assay was performed with 5 replicates in 3 independent experiments.

### Statistical Analysis

All statistical analysis was performed with SPSS 16.0 (SPSS Inc., Chicago, IL, USA). The classical t-test method was used to compare the data between the two groups that conformed to normal distribution and p<0.05 was considered statistically significant.

## Results

### SIPS Model of DLBCL Is Successfully Induced by 30 μM tBHP

**Figure 1:**
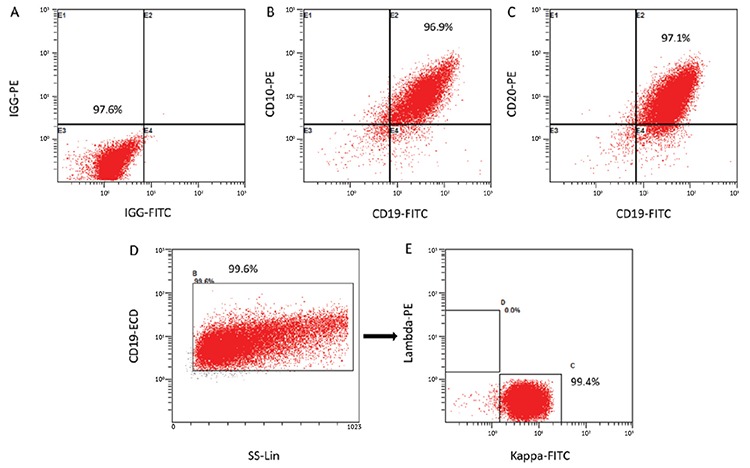
Immunophenotype of DLBCL LY8 cell lines. LY8 cell line was used for detecting immunophenotyping by flow cytometry (FCM). (A, B) FCM analysis of CD19, CD20, and CD10 expressions in LY8 cell line. (C) Negative control of PE and FITC molecules. (D) FCM analysis of type of immunoglobulin light chain in LY8 cell line.

**Figure 2:**
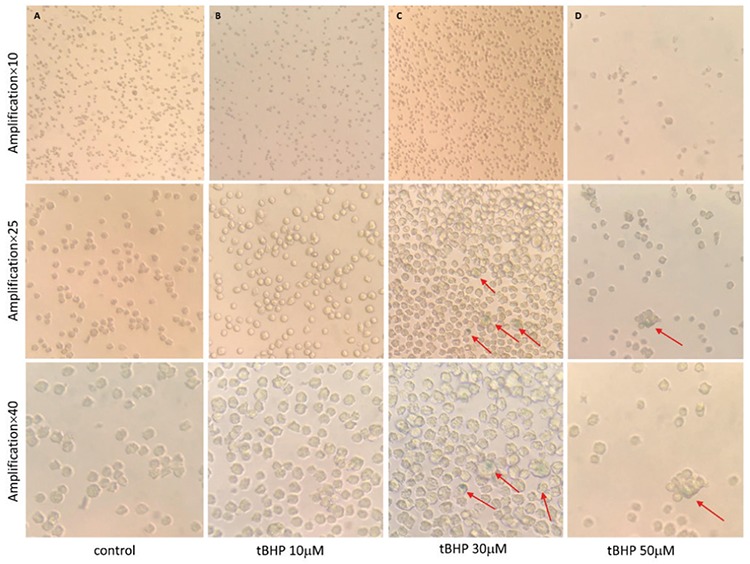
Stress-induced premature senescence model of DLBCL induced by 30 μM tBHP for 24 h. LY8 cells were treated with 10, 30, and 50 μM tBHP respectively for 24 h in vitro. Then cells were stained for β-galactosidase. A represents the control group, which was observed at amplifications of 10x, 25x, and 40x. B represents the 10 μM tBHP group, which was observed at amplifications of 10x, 25x, and 40x. C represents the 30 μM tBHP group, which was observed at amplifications of 10x, 25x, and 40x. D represents the 50 μM tBHP group, which was observed at amplifications of 10x, 25x, and 40x.

The immunophenotype of the DLBCL LY8 cell line was CD19-, CD20-, and CD10-positive and the immunoglobulin light chain is the kappa type (Figure 1). The LY8 cells were respectively stimulated with 10, 30, and 50 µM tBHP for 24 h in vitro and treated with senescence β-galactosidase staining (Figure 2). Compared with the control group, cell growth was obviously affected by tBHP intervention. Senescent DLBCL cells had enlarged nuclei, irregular shapes, and clumps of growth (Figure 2C). Stimulation with 50 µM tBHP led to a large amount of apoptosis in LY8 cells (Figures 2D). On the other hand, there were no senescent cells in the 10 µM and 50 µM tBHP groups (Figures 2B and 2D), but blue-green senescent cells could be obviously observed in the 30 µM tBHP group (Figure 2C). These results suggest that the SIPS model of DLBCL can be successfully induced by 30 µM tBHP for 24 h in vitro.

### SIPS Activates *SENEX* and the p16/Rb Pathway

**Figure 3:**
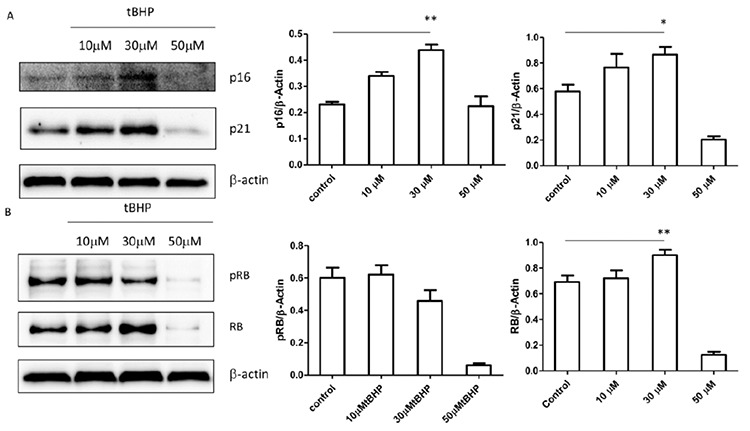
Stress-induced premature senescence activates the p16/Rb pathway. LY8 cells were treated with 10, 30, and 50 μM tBHP respectively for 24 h in vitro. After 48 h they were harvested for western blot analysis. (A) The expression of p16 and p21 protein in LY8 cells under the pressure of tBHP. (B) The expression of Rb and pRb in LY8 cells under the pressure of tBHP.

Both p21 and p16 are important markers of cellular senescence [17]. In our studies, we observed that the expression of p21 protein was significantly increased in the 30 µM group compared with the control group (p<0.01) (Figure 3A), and the level of p21 was also obviously upregulated in the 30 µM group compared to the control group (p<0.05) (Figure 3A). These results indicate that senescence promoted p16 and p21 activation.

Studies indicated that the Rb pathway inhibits transcription of genes that are necessary for the transition from the G1 to the S phase. Central to this pathway is the regulation of phosphorylation of the Rb protein [18]. In our studies, we found that the expression of the Rb protein was higher in the 30 µM group than the control group (p<0.01) (Figure 3B). In contrast, the level of pRb expression in the 30 µM group was downregulated compared with the control group (Figure 3B). These results indicated that senescence inhibited the phosphorylation of Rb.

**Figure 4:**
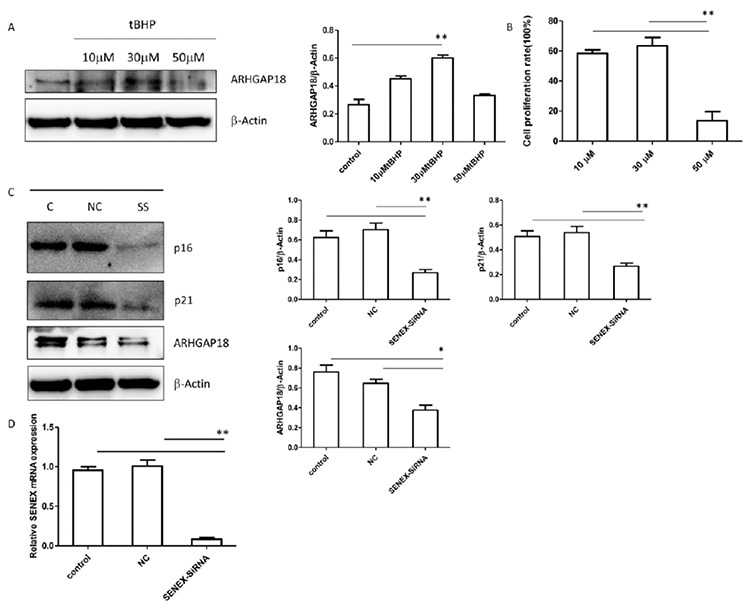
Stress-induced premature senescence promotes *SENEX* activation and proliferation and *SENEX* activates the p16 pathway. (A) LY8 cells were treated with 10, 30, and 50 μM tBHP respectively in vitro. After 48 h they were harvested for western blot analysis. The expression of *SENEX* in LY8 cells is shown under the pressure of tBHP. (B) LY8 cells were transfected with *SENEX*-SiRNA or NC in vitro. After 48 h they were harvested for western blot analysis. The expression of p16 and p21 in LY8 cells transfected with *SENEX*-SiRNA/NC is shown (C = unprocessed control group; NC = LY8 cells transfected with NC group; SS = LY8 cells transfected with *SENEX*-SiRNA group). (C) LY8 cells were treated with 10, 30, and 50 μM tBHP respectively for 24 h in vitro. After 48 h they were harvested for western blot analysis. The cell proliferation rates of LY8 cells induced by tBHP were measured with CCK8 analysis. **p<0.01.

Based on these results, we further tested the expression of ARHGAP18, which is encoded by the *SENEX* gene, in the DLBCL cellular senescence model. The expression of ARHGAP18 was significantly higher in the 30 µM group than the control group (p<0.01) (Figure 4A). This suggests that ARHGAP18 was significantly increased in senescent DLBCL cells. The relationship between senescence, the *SENEX* gene, and the p16/Rb pathway needs further exploration.

### SIPS Promotes Proliferation

In this study, the cell proliferation rate was detected respectively by CCK8 analysis in the 10 µM tBHP group, 30 µM tBHP group, and 50 µM tBHP group. We found that the cell proliferation rate in the 30 µM tBHP group was significantly upregulated compared with the 50 µM tBHP group (p<0.01) and was also higher than in the 10 µM tBHP group (Figure 4B). Proliferation of senescent DLBCL cells is accelerated compared with other cells under the pressure of tBHP. These results suggest that SIPS promotes proliferation.

### *SENEX* Activates the p16 Pathway

To determine whether *SENEX* is important in the activation of the p16 pathway, LY8 cells were transfected with the individual small interfering RNA target *SENEX* gene (*SENEX*-siRNA) and scrambled negative control siRNA (NC) to silence the *SENEX* gene, and then we analyzed the expression of p16/p21. We verified the efficiency of transfection at both gene and protein levels. The level of ARHGAP18 was obviously reduced in the *SENEX*-SiRNA group compared to the control and NC groups (Figure 4C), and the expression of *SENEX* mRNA was also significantly decreased in the *SENEX*-SiRNA group compared with the control group (LY8 cells without any treatment) and the NC group (Figure 4D). These results suggest that transfection with *SENEX*-SiRNA could silence the *SENEX* gene in DLBCL cells. The expression of p16 was significantly decreased in the *SENEX*-SiRNA group compared with the NC and unprocessed groups (Figure 4C). Consistent with this, the expression of p21 was also decreased in the *SENEX*-SiRNA group compared with the NC and unprocessed groups (Figure 4C). These results suggest that *SENEX* activates the p16 pathway in DLBCL.

## Discussion

Cell senescence is a state of cell cycle arrest under stress, which is an indispensable mechanism to prevent the proliferation of injured cells [19]. The permanent growth arrest caused by cell senescence suggests that the senescence response can inhibit the development of tumors. It is now thought that senescence-induced growth retardation is irreversible because no physiological stimuli have been found to enable senescent cells to reenter the cell cycle [20]. However, when cells undergo some molecular biological changes, such as the successive inactivation of certain tumor-suppressor genes, it can cause abnormal proliferation of senescent cells. Senescence-induced inhibition of proliferation is strictly irreversible. It is supported and maintained by at least two major tumor-suppressor gene pathways (p53/p21 and p16INK4a/pRb signaling pathways) [21]. In addition to growth arrest, senescent cells also exhibit a wide range of alterations in chromosome and gene expression. This will lead to some proinflammatory cytokines, chemokines, growth factors, and protease and other cell secretory factor secretion changes, and the abnormal expression of this factor is known as the senescence-related secretory phenotype or senescence-associated secretory phenotype (SASP). SASP, associated with a large number of paracrine activities, will have a wide range of effects on cells and the whole body [22]. On the one hand, it can inhibit the development of tumors and promote tissue repair and regeneration in the face of injury. On the other hand, these abnormal secreted cytokines can cause malignant transformation of normal cells and promote the occurrence and development of tumors [23].

In this study, we investigated tBHP-induced SIPS in a DLBCL cell line. First of all, the immunophenotype of the LY8 cell line is CD19+, CD20+, and CD10+, which is a phenotype consistent with lymphocytes of germinal center origin (Figure 1). Senescent cells usually become larger in size and express β-galactosidase with high enzymatic activity at pH 6 [24]. After stimulation with tBHP, LY8 cells were treated with senescence β-galactosidase staining. Compared with the control group, we observed that cell growth was obviously affected by tBHP intervention. Senescent cells could be most obviously observed in the 30 µM tBHP group. The senescent cells had enlarged nuclei, irregular shapes, and clumps of growth (Figure 2). These results suggest that SIPS in DLBCL cells can be successfully induced by 30 µM tBHP. Secondly, the cell proliferation rate was detected by CCK8 analysis. We found that cell proliferation rate was significantly upregulated in the 30 µM tBHP group. Cell proliferation of senescent DLBCL cells is accelerated compared with other cells under the pressure of tBHP (Figure 4). These results suggested that SIPS promotes the proliferation of DLBCL cells.

Senescence is mediated through the p53 pathway, which transactivates the cyclin-dependent kinase inhibitor p21, or through the p16 pathway to inhibit cyclin-dependent kinases 2 and 4, preventing phosphorylation of the Rb protein [26,27]. In our studies, we observed significantly upregulated p21 and p16 protein in senescent cells. These studies suggest that senescence is characterized by developmental cues that converge on p21 and p16 proteins. Studies indicated that the Rb pathway inhibits the transcription of genes that are necessary for the transition from the G1 to the S phase. Central to this pathway is the regulation of phosphorylation of the Rb protein [28]. In our studies, we found significantly high expression of Rb protein. This indicates that the p53/p21/Rb and p16/Rb axes are both important signaling pathways involved in the induction of senescence.

The *SENEX* gene has been proved to provide a unique gatekeeper function in the SIPS and apoptosis pathways in ECs [13]. Furthermore, *SENEX* overexpression induced an increase in both the mRNA and protein levels for p16 and there was a decrease in the protein expression of the hyperphosphorylated Rb. These results indicated that *SENEX* activated the p16/pRb pathway [13]. In our study, the expression of *SENEX* protein was significantly higher in the 30 µM group than the control group. It is suggested that the *SENEX* protein was significantly increased in senescent DLBCL cells.

## Conclusion

We investigated a SIPS model of DLBCL and found that it can be successfully induced by tBHP. SIPS formation activates the *SENEX* gene and the p16/Rb pathway and promotes DLBCL cell proliferation. Furthermore, *SENEX* activates the p16 pathway in DLBCL. SIPS promotes proliferation by activating *SENEX* and the p16/Rb pathway in DLBCL. *SENEX*-related SIPS may serve as an important target for relapsed/refractory DLBCL therapy. By improving the knowledge on the molecular basis of senescence, novel strategies relying on senescence induction will reach the clinic as potential cancer therapies. *SENEX*-related senescence may serve as an important target for relapsed/refractory DLBCL therapy.

## Figures and Tables

**Table 1:**
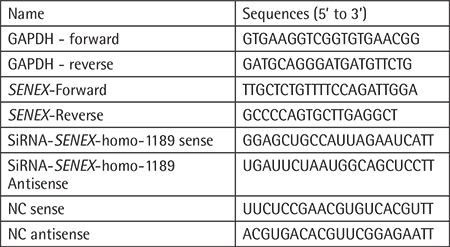
Sequences used for quantitative real-time polymerase chain reaction primers and SiRNA transfection.

**Table 2:**
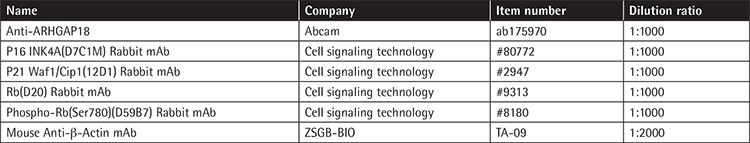
Primary antibodies used for western blotting.
